# A machine learning framework for predicting fuel consumption and CO_2_ emissions in hybrid and combustion vehicles: comparative analysis and performance evaluation

**DOI:** 10.1371/journal.pone.0342418

**Published:** 2026-02-11

**Authors:** Rania A. Ibrahim, Nahla E. Zakzouk

**Affiliations:** Electrical and Control Engineering Department, College of Engineering and Technology, Arab Academy for Science and Technology, Alexandria, Egypt; Tishk International University, IRAQ

## Abstract

Accurate estimations of fuel consumption and carbon emissions insights are critical for performance benchmarking, emissions compliance, and the optimization of energy management strategies in vehicles’ systems. Unlike model-based predictive approaches that require complex modelling, machine learning (ML) predictive models learn patterns directly from data, w making them flexible, automated, and scalable solutions for complex nonlinear systems that can easily adapt to diverse sets of data with high predictive accuracy. These models typically span from linear and nonlinear models to ensemble approaches, where the latter are often preferred owing to their ability to aggregate multiple learners and more effectively capture intricate relationships.. This study develops a predictive ML framework for estimating vehicle emissions and fuel consumption in lightweight vehicles via a real-world dataset. The primary contribution of this work lies in the fusion and integration of internal combustion engine vehicle (ICEV) and plug-in hybrid vehicle (PHEV) datasets into a common modelling workflow, whereas most existing studies rely solely on combustion-vehicle datasets only. Another contribution is the dual-forecast capability of the proposed model, enabling simultaneous prediction of both vehicle emissions and fuel usage rather than solely predicting emissions, as in most prior studies. In contrast, this study offers a unified framework capable of accurately forecasting both vehicle emissions and energy consumption. The adopted broader and more diverse mixed dataset enhances generalization, in addition to making the proposed model a practical and reliable tool for environmental assessment, sustainable vehicle development, and policy decision-making.

## 1. Introduction

The transportation sector continues to play a pivotal role in influencing global energy demand and climate trajectories. With over 1.4 billion global vehicle fleets worldwide, road transport adds consistent pressure on industries and governments to decarbonize mobility systems, reduce their energy consumption and increase their energy efficiency. According to the Energy Institute Statistical Review of World Energy for 2023–2024, the transport industry represents approximately 28% of global final energy use and nearly 16% of total global greenhouse gas emissions, with light-duty vehicles being the primary contributors [1,2]. In fact, vehicle fuel consumption is a significant economic index, while vehicle carbon emissions have a critical impact on the environment [3]. Hence, accurate prediction of these two variables is vital to ease environmental policy formulation, reduce unnecessary fuel usage, support cost-effective decision-making in infrastructure planning and vehicle design, and commit to sustainable development goal requirements [4,5].

As the world accelerates toward clean sustainable energy, transportation electrification is becoming a critical driver of environmental progress, economic innovation, and energy resilience. Hence, comparative analysis between internal combustion engine vehicles (ICEVs) and hybrid electric vehicles (HEVs) in terms of energy usage and emissions is increasingly essential for guiding policy decisions, infrastructure development, and consumer choices. While ICEVs still dominate the global fleet—particularly in emerging economies and regions with limited access to electrical charging infrastructure—they continue to offer perceived advantages in terms of upfront costs, consumer acceptance, and well-established fuelling service networks. However, these benefits are gradually outweighed by the long-term benefits associated with HEVs [6].

HEVs use a combination of an internal combustion engine and an electric motor. This enables HEVs to feature less noise, reduce fuel consumption and costs and, in turn, lower greenhouse gas emissions, which in turn position them as a more viable, clean and secure promising alternative. Moreover, HEVs can capture and reuse energy while braking through regenerative braking systems, thus enhancing efficiency and reducing wear on brake components. Additionally, from a cost perspective, HEVs’ lower maintenance costs are noteworthy, with additional revenues to HEV owners when they are participating in primary frequency regulation markets [7]. These advantages make HEVs a practical transitional technology toward full vehicle electrification [8].

Nevertheless, HEV energy consumption and accurate emission prediction are not easy because they depend on several dynamics and interrelated variables. For this reason, several approaches exist that can be divided into a) traditional physical modelling approaches and b) data-driven approaches [9]. In predictive physical modelling approaches, fuel consumption can be computed from engine data along with engine cycle conditions and vehicle technical parameters [10]. The designed model is built from mathematical formulas or empirical models using variables such as vehicle component-related variables, vehicle dynamics variables, and traffic- and environment-related variables [11,12]. Additionally, statistical models tend to estimate model parameters by finding a relationship between independent variables and dependent variables, requiring prior determination of this relationship, which may limit the model’s accuracy [13]. Although modelling-based approaches require less data, they require deep knowledge and details of the input parameters, which could be computationally intensive. Additionally, model parameters must be identified to achieve a high degree of prediction accuracy, making this approach more applicable to fixed-route scenarios, thereby limiting their generalizability [9]. Furthermore, since this approach requires a high degree of knowledge of the physical model, its scope of application is somewhat limited, making it challenging to apply the same model to diverse vehicle technologies [14].

Data-driven approaches, on the other hand, have become increasingly popular for predictive analysis of vehicle energy usage and emission estimations because of their ability to handle large amounts of data with a high degree of accuracy. They rely on on-board equipment and embedded sensors that gather vast amounts of vehicle information and data related to fuel consumption and vehicle emissions. These datasets are then used to train models that estimate actual fuel consumption and emissions by identifying patterns in the data and accordingly making predictions [15]. Although data-driven algorithms are proven for their high efficiency and improved accuracy, their performance is highly dependent on data quality, and they are often susceptible to overfitting, particularly when trained on small datasets. Thus, large datasets are essential for accurate predictions [16].

While modelling-based approaches often struggle in capturing nonlinear dependencies, exhibit limitations in scalability with large data volumes, and have difficulty representing complex interactions between features, ML-based data-driven models stand out as more effective alternatives [13]. Designed to learn from large data volumes, ML algorithms can capture nonlinear behaviour, identify patterns, and correlate variables in an effective manner by training on labelled data to predict specific dependent variables via independent features. Among ML models, regression-based prediction algorithms are commonly utilized, ranging from simple linear regression to complex nonlinear models [17]. Nonlinear algorithms such as support vector regression (SVR), decision trees (DTs), and Gaussian processes (GPs) [18], tend to have high predictive accuracies and no assumption of linearity, are more robust to outliers, and are more adaptable to large and scaled data than linear algorithms are. More advanced ensemble ML models, such as random forest (RF), gradient boosting (GB), bagging and their consecutive variants, achieve superior performance by aggregating the outputs of multiple learners, thus enhancing the overall model generalizability [19–21]. Additionally, deep learning (DL) techniques are promising algorithms with superior performance and minimal manual feature engineering [22]. Despite their prominent performance, DL model architectures require large amounts of computational resources and more complex implementations [23]. Thus, they are more favourable in highly complex applications and datasets, such as those related to time series analysis and high-dimensional feature spaces, where other algorithms may fall short [24].

Notably, electric vehicles (EVs) play an increasingly important role in sustainable transportation and represent a rapidly expanding segment of modern mobility because of their zero tailpipe emissions and high energy efficiency [25]. However, this study focuses on ICEVs and HEVs since EVs produce no tailpipe emissions, whereas the primary objective of this study is to forecast fuel consumption and exhaust emissions—parameters that apply only to combustion-based powertrains. Moreover, the real-world dataset used in this work includes data for only these two vehicle types, with no EV entries available. Accordingly, the analysis focuses on vehicle types for which both emissions and fuel consumption are measurable and relevant.

In this paper, based on comprehensive datasets provided by the Natural Resources Canada (NRCan) for light-duty vehicles marketed in Canada in [26], different ML-based predictive models for vehicle fuel consumption and carbon emissions are developed. Compared with other predictive models developed in related works that use datasets from the same source, the proposed RF-based models feature key novel contributions that can be clearly itemized as follows:

- **Dual-output prediction**: Prediction of both fuel usage and CO₂ emissions. Most studies address only emissions.
- **Fusion of datasets**: A unified comprehensive dataset is formed that combines ICEV and HEV data, providing a broader generalizable model.
- **Comprehensive model Evaluation:** Compares 13 ML algorithms (linear, nonlinear, and ensemble ML models) to select the one with the highest accuracy and best generalizability.
- **Simplified architecture with high accuracy:** the proposed framework achieves performance comparable to that of DL models but with a simpler architecture and lower computational cost.

Thus, unlike prior work limited to single-technology datasets or emission-only predictions, this study uniquely introduces a dual-target, ensemble-based ML framework for both ICEVs and PHEVs and offers superior generalization capabilities with reduced computational costs and model implementation complexity compared with those of DL-based counterparts.

The structure of this paper is divided into seven sections. Following the *Introduction*, the second section includes a *Literature Review* on different forecast techniques in the field of vehicles. The third section is dedicated to *NRCan dataset-based related work,* followed by the *Methodology* section, which explains the proposed approach framework in detail. The fifth section includes *Results and Performance Evaluation* for both fuel consumption and emissions forecast tasks. A *Discussion* is presented in the sixth section, involving interpretation of the results and performance insights of the proposed approach, in addition to its main contributions and future perspectives. Finally, the last section includes the main *Conclusion* drawn in this study.

## 2. Literature review

Vehicle energy consumption and emission estimation models have been the topic of extensive studies and have been the focus of many researchers. For fuel consumption estimation, models are usually either analytical or statistical and incorporate both internal vehicle parameters and the environmental and traffic conditions that influence energy usage [27]. Analytical approaches concentrate on the operation features of the vehicle, such as engine parameters and output power, whereas statistical approaches rely on vehicle activity and the statistical attributes of fuel consumption data [28]. With respect to emissions, several well-established physical models exist that rely on lookup tables and microscale modelling for emission output estimations. These models estimate emissions on the basis of look-up tables and data obtained from dynamometer data related to vehicle acceleration and speed [29,30]. Some models may incorporate vehicle-specific parameters for emission calculations, whereas others use functions of the mean travel speed throughout a complete driving cycle to calculate greenhouse gas emissions [31].

With respect to all the aforementioned techniques, while these approaches offer high relevance related to traffic simulation, they often rely on predefined functional forms, require extensive calibration, or lack adaptability to different terrains and driving conditions. Moreover, they neglect some specific vehicle-oriented characteristics, such as the engine size, manufacturer and engine model. These shortcomings have paved the way for data-driven artificial neural network (ANN)- and ML-based techniques. These techniques can automatically learn complex, nonlinear relationships from data and can provide accurate estimates from large datasets with high levels of generalizability and adaptability without the need for extensive modelling or analysis [32,33].

In [34], an ANN-based predictive model was applied for fuel consumption analysis in motor vehicles, especially passenger cars. The MLP (multilayer perceptron) 22--10--3 network was selected from the created neural networks, which yielded high prediction accuracy, with an R^2^ of more than 0.98. In [35], a predictive analysis model for fuel consumption and carbon dioxide emissions was proposed for light-duty vehicles via ML and statistical methods. Among the latter, the univariate polynomial regression model achieves the highest R^2^ values in predicting emissions and fuel consumption. The work in [36] explores two predictive models, i.e., the RF and ANN, which were trained on data from transient vehicle tests, with a specific focus on instantaneous fuel consumption. Although the ANN model was better at predicting fuel consumption than the simpler RF model was, the improvement in its model fitness came at a considerable expense in terms of CPU time. This amounted to a few minutes versus several hours for the RF and ANN models, respectively, which proves that the implementation complexity and computational burden of the ML-based models are lower than those of the ANN models. In [37], a rule-based control method along with an ML-based estimation technique was used to improve fuel consumption efficiency and reduce carbon emissions in hybrid EVs. Seven ML methods to estimate fuel consumption were tested, i.e., RF, REP Tree, M5P, random tree, multilayer perceptron, linear regression, and decision jump, among which RF achieved the highest accuracies of 97% and 90% for two different test datasets. On the other hand, in [38], a parallel hybrid electric vehicle was modelled, and a new AI rule-based and battery-priority control method was proposed to reduce fuel consumption and carbon emission values to minimum values. Finally, in [39], a digital twin trained with ML algorithms was applied to emission tests of a hybrid vehicle where the RF model demonstrated high accuracy in predicting CO_2_, THC, and CH_4_ emissions, with R² values close to 1.

Notably, optimization techniques are also widely used for vehicle parameter estimation via evolutionary and metaheuristic algorithms [40]. These techniques can be used to identify battery parameters, improve model accuracy, and enhance vehicle performance in areas such as energy consumption, charging strategies and energy coordination [41]. In [42], a gradient-based optimizer was applied for parameter identification of an EV battery model, whereas a genetic algorithm (GA)-based energy management strategy was proposed in [43] for fuel cell hybrid electric vehicles. Particle swarm optimization (PSO) is known for its effectiveness in handling nonlinear and multiobjective problems; thus, it was applied in [44] for estimating equivalent circuit model parameters in EVs. However, gray wolf optimization (GWO), which provides robust global search capabilities, can be applied for accurate parameter identification in EVs [45]. On the other hand, the modified Walrus optimization (MWO) algorithm has been successfully used in parameter estimation for the proton exchange membrane (PEM) of fuel cell-based EVs, significantly increasing prediction accuracy [46]. The MWO algorithm, as an advanced metaheuristic optimizer, efficiently explores the parameter space to identify the optimal set of system parameters that minimize prediction errors. Compared with the standard Walrus algorithm, its modifications improve convergence speed and global search capability, making it particularly effective for the complex, nonlinear models typical of hybrid powertrains. Finally, a multiobjective optimization framework is introduced [47] for improving internal combustion engine performance in HEVs, specifically by minimizing fuel consumption and emissions.

Notably, although this study focuses on ICEVs and HEVs, similar ML and optimization approaches have been successfully applied to other clean technologies, including fuel cell vehicles (FCVs). For example, the parameter estimation techniques used in HEVs can be adapted to proton exchange membrane (PEM) fuel cells, as demonstrated in [48]. These methods effectively capture nonlinear system behavior and enable reliable prediction of system performance, indicating the potential for extending these models to diverse vehicle technologies.

## 3. NRCan dataset-based related work

Since this work is based on a publicly available dataset for different types of vehicle technologies offered by the NRCan, which can be found in [26], Table (1) compares studies that utilized the same dataset in estimating fuel consumption or/and CO_2_ emissions with the method proposed in this study. Further details of the dataset are discussed further in Section 3 Methodology. As observed from Table (1), several studies have been conducted using the same data source offered by the NRCan, although as reported, analysis varies in terms of temporal coverage, modelling objectives and type of engines used in the study. These studies illustrate how the dataset has been employed to support a range of predictive tasks, using diverse ML and statistical approaches, thus reflecting the broad applicability of the NRCan dataset and its flexibility in several investigations related to vehicle energy consumption and emissions.

**Table 1:** NRCan dataset-based related work for fuel consumption and CO_2_ estimation.

Ref	Dataset Span	Number of Data points	Prediction Technique with highest accuracy	Predicted Objective		Dataset	
				Consumption	CO_2_ Emissions	ICEV	PHEV
[49]	1995–2022	7385	Ensemble ML	x	✓	✓	x
[50]	Not specified	5085	Ensemble ML	x	✓	✓	x
[51]	2017–2021	7384	Ensemble ML	x	✓	✓	x
[52]	2014–2023	Not specified	Ensemble ML	x	✓	✓	x
[53]	2017–2021	7385	Ensemble ML	x	✓	✓	x
[54]	2014–2020	7384	Ensemble ML	x	✓	✓	x
[55]	Not specified	Not specified	ANN	x	✓	x	✓
[35]	2017–2021	4973	DL	✓	✓	✓	x
[56]	Not specified	6281	DL	x	✓	✓	x
[57]	2017–2021	7385	DL	x	✓	✓	x
[58]	2017–2021	7385	DL	x	✓	✓	x
Proposed	1995–2025	7737	Ensemble ML	✓	✓	✓	✓

With respect to the applied predictive data-driven approach in each study that achieved the highest performance, ensemble ML was applied in [49–54], ANN was used in [55], and DL was used in [35,56–58]. These studies, which applied datasets from the same source, vary in their accuracy and performance, as will be discussed later in Section 5. On the other hand, with respect to the predicted variable, almost all works focused exclusively on CO₂ emission prediction, neglecting fuel and energy consumption, which was only studied by [35]. Finally, almost all these studies depended solely on ICEV datasets, whereas [55] used only PHEV datasets. Therefore, as shown in Table 1, the approach proposed in this paper outperforms its counterparts because of its generalized ability to predict both vehicles’ carbon emissions and fuel consumption based on combined datasets of both ICEVs and PHEVs. Moreover, it achieved high accuracies yet a less complex architecture than the DL-based models proposed in the literature, as will be demonstrated in section 5.

## 4. Methodology

This paper focuses primarily on analysing two combined datasets for light-duty vehicle categories sourced publicly and provided by NRCan covering both ICEVs and PHEVs [59]. The primary objective is to evaluate and compare the performance of a range of regression models in accurately forecasting two target variables, energy consumption and CO_2_ emissions, across different vehicle types and based on a wide range of vehicle characteristics as inputs to the model to determine the one with the highest accuracy. These input attributes consist of a combination of numerical features such as engine size, number of cylinders, and combined fuel-consumption rating, as well as categorical features such as Make, vehicle class, transmission type, and fuel type.

The analyses carried out in this work, including modelling, preprocessing, testing, evaluation and visualizations, are implemented via Python programming within the Google Colab environment. Many computing libraries, such as NumPy and Pandas for data handling, Matplotlib for visualization, and scikit-learn for preprocessing, pipeline construction, cross-validation, and classical ML models, are used. In addition, the libraries for state-of-the-art algorithms such as XGBoost, LightGBM, CatBoost, and HistGradientBoosting were imported and configured directly within the code. Predictive algorithms were selected on the basis of algorithm diversity, capturing different learning approaches, popularity with proven performance in the ML community, and computational efficiency, thus covering three main domains of data-driven regression analysis. The explored domains are a) linear regression, b) nonlinear regressors, and c) ensemble ML models. The selected algorithms are suitable for structured datasets, with the specific objective of identifying the model with the highest predictive accuracies while maintaining a high level of interpretability and low computational complexity.

**Fig 1:**
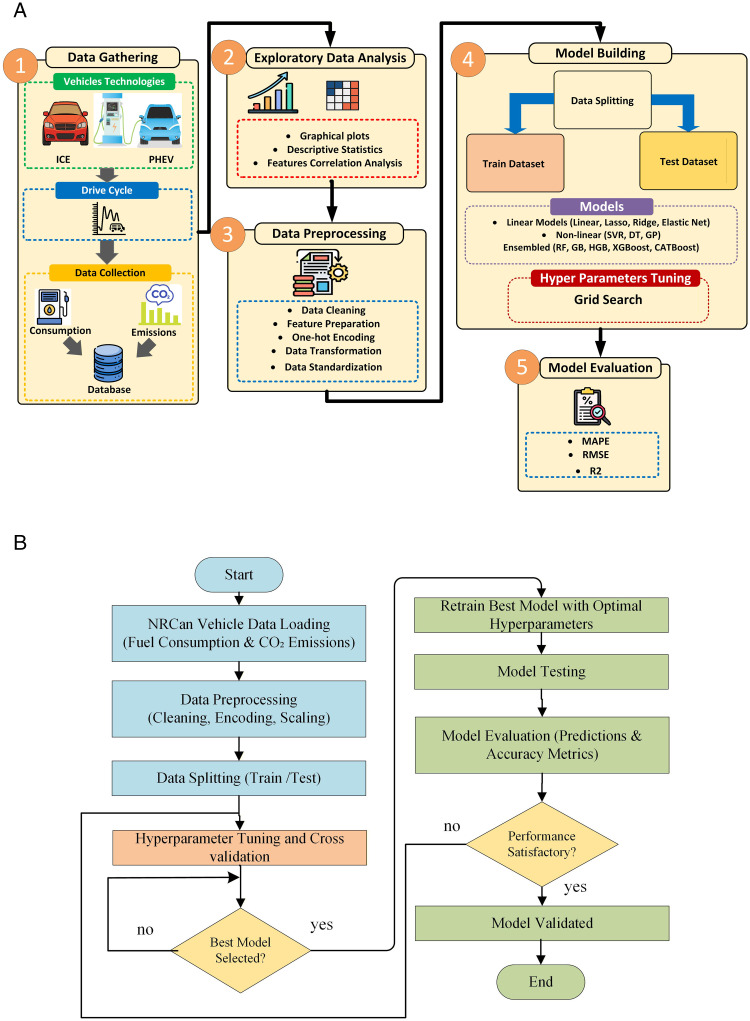
Methodology (A) Overall framework, (B) flowchart.

The analytical stages of the proposed framework are depicted in Fig 1. As shown in Fig 1(A), the framework follows a series of sequential steps, starting with data collection and exploratory data analysis, where the combined ICEV and PHEV datasets are gathered, inspected, and statistically analysed to understand feature distributions and correlations. This is followed by a data preprocessing phase, which includes data cleaning, handling of missing values, categorical feature encoding, numerical feature scaling, and dataset splitting to ensure model readiness for the subsequent processing stage. The next stage is the model building stage, where a diverse set of ML models are trained via the aforementioned pipeline. Hyperparameter optimization is carried out via GridSearchCV with 5-fold cross-validation to ensure model robustness and predictive error consistency. Finally, the model evaluation stage assesses the predictive performance of all the tested models via multiple statistical indices, as well as visual diagnostic parity plots and statistical significance testing. Fig 1(B) further explains the workflow in a flowchart form to demonstrate the logical progression between the different analytical stages of this study.

### 4.1 Data gathering and collection

The publicly accessible datasets provided by NRCan have been consistently releasing annual performance statistics for light-duty cars in Canada for more than ten years. Covering a wide range of vehicle technologies available in the market, NRCan regularly updates these datasets, with corresponding information on several technical aspects, such as vehicle manufacturers and models, engine size, fuel type, and transmission system type. In addition, emissions and consumption data are also provided in the dataset, which are derived through a 5-cycle standard approved by the Government of Canada. Using a chassis dynamometer, the derived data for fuel consumption and CO₂ emissions for drivetrains are collected following controlled laboratory protocols to ensure comparability across vehicle technologies and model years. Unlike European standards such as the ECE-15, the EUDC, and the combined NEDC, which are somewhat modal and simplified, the NRCan standard driving cycles incorporate a broader range of driving conditions, including city and highway driving, cold temperatures, air-conditioning loads, and high-speed/aggressive behaviour, making them a credible dataset and ideal foundation for developing predictive studies [60]. NRCan can compile and validate this information before the release of annual datasets, making it one of the most comprehensive and credible sources for fuel consumption and emission benchmarking in Canada.

As mentioned earlier, the compiled “Fuel consumption rating” open databases are obtained from the official website of the Canadian Ministry of Transportation, where two datasets (for ICE and PHEV) are measured to provide specific vehicle information using mixed data types of both numerical and categorical categories and encompassing vehicle information, including the make, model, engine details, transmission type, fuel consumption rates, and CO_2_ emissions [61]. These vehicles usually weigh 10,000 pounds or less and are primarily used for transporting passengers and cargo, including cars, vans, sport utility vehicles (SUVs), and pickup trucks. The original compiled data for consumption and CO_2_ emissions were collected from vehicle manufacturers through laboratory standardized testing procedures, thus simulating real-world driving scenarios [35].

In this work, the authors combined two sets of ICEV and PHEV data from 2017--2025, forming one dataset containing 11 attributes and 7737 records, which differentiates this work from other related works in the literature by offering detailed insights into vehicles presently available in the Canadian market, covering both traditional combustion and electric vehicles. This merging provides a more comprehensive and holistic view of policy decisions and network planning to determine total fuel demand, which is fundamental for transportation infrastructure development, as well as identifying the level of carbon emissions produced by both vehicle categories to verify the role of PHEVs in fulfilling sustainability goals.

### 4.2 Exploratory data analysis

Dataset exploration is a vital step for data understanding and preparation before analysis; thus, data analysis, data visualization and correlation analysis are carried out in this subsection. Table 2 presents the descriptive statistics of the numerical features of the studied dataset, which facilitates understanding the data distribution and serves as an indicator of dataset quality. On the basis of the data provided, the average total fuel consumption is 10.639 L/100 km, of which 12.4 L/100 km are from the city, whereas the remaining 9 L/100 km are from highways. Additionally, the average automobile CO_2_ emissions are 243.833 g/km, with a corresponding standard deviation of 66.2 g/km. Furthermore, Table 2 illustrates a wide range of existing engine sizes, ranging from 0.6 litres to 8.4 litres, which are expected to be significant factors influencing both emissions and consumption prediction. In addition, CO_2_ experiences large variations, reflecting the existence of both efficient and high-emission vehicles due to the fusion of the ICEV and EV datasets. Moreover, the standard deviation (std) indicates that the expected value distributions of both total consumption and emissions are well defined, thus enabling accurate forecasting.

**Table 2:** Descriptive statistics of the combined datasets.

	Count	mean	std	min	25%	50%	75%	Max
**Engine_size**	7736	3.12	1.35	0.60	2.00	3.00	3.70	8.40
**Cylinders**	7736	5.57	1.83	2.00	4.00	6.00	6.00	16.00
**fuel_consumption_city**	7736	12.41	3.56	4.20	9.90	11.90	14.50	30.60
**fuel_consumption_hwy**	7736	9.00	2.23	4.00	7.40	8.70	10.20	20.60
**fuel_consumption_comb**	7736	10.64	3.23	1.80	8.70	10.40	12.50	26.10
**co2_emissions**	7736	243.83	66.20	14.00	202.00	242.00	286.00	522.00

**Fig 2:**
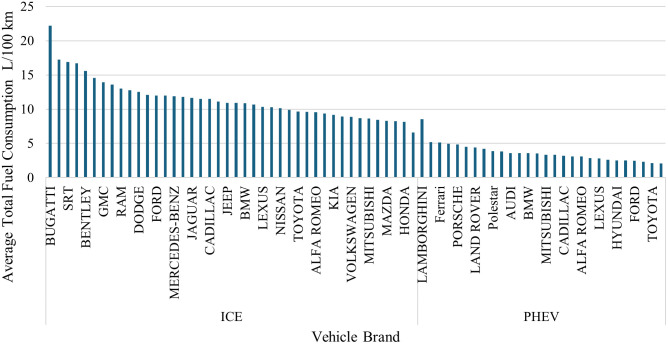
Average fuel consumption per vehicle brand.

**Fig 3:**
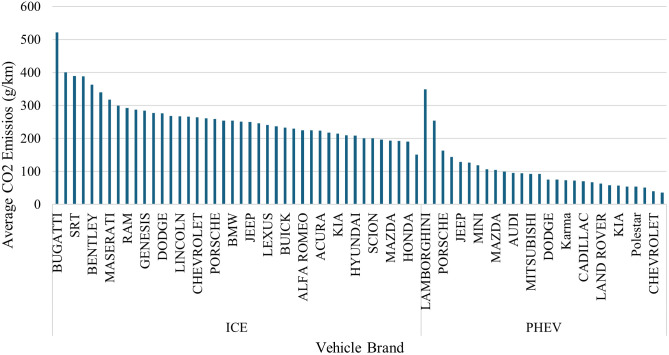
Average CO_2_ emission per vehicle brand.

Fuel consumption and CO_2_ emissions are presented in Figs 2 and 3, respectively. Luxury and high-performance ICEV vehicles have the highest energy demand and emission profile among all brands. High-performance ICEV vehicles are the highest among all brands in terms of energy demand and emission profile.

**Fig 4:**
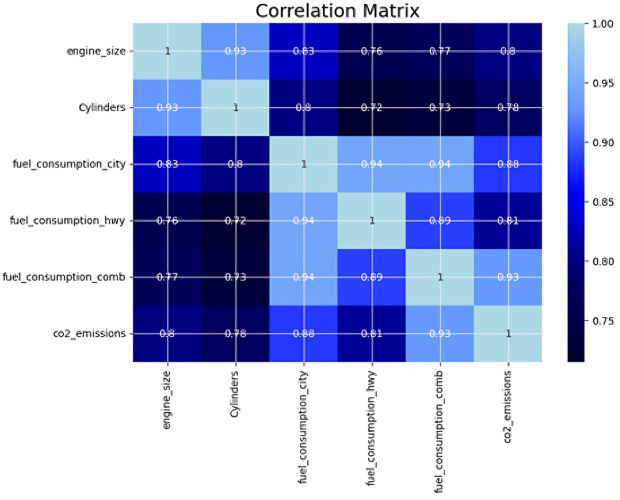
Heatmap of quantitative independent variables.

A correlation heatmap using Pearson’s correlation coefficients is presented in Fig 4 to visualize the relationships between features and help identify feature combinations with strong correlations among all the quantitative numerical variables, such that all these variables range from highly positive to highly negative. Since fuel consumption and CO_2_ emissions are the dependent variables, it is concluded from this heatmap that fuel consumption in both cities (fuel_consumption_city) and highways (fuel_consumption_hwy) is highly correlated with combined fuel consumption (fuel_consumption_comb) as well as CO_2_ emissions (co2_emissions), followed by engine size (engine_size) and cylinder (cylinders). Similarly, there is a strong positive relationship between the same variables and CO_2_ emissions. Additionally, some other general observations could be drawn from the feature map, such as that larger engines and more cylinders usually imply higher fuel consumption and emissions. Additionally, the observed strong correlations among several numerical predictors indicate the presence of multicollinearity, which may be useful for the selected ML models to learn their joint influence on fuel consumption and CO₂ emissions. Furthermore, the observed scale difference between correlated variables suggested the use of standardization in the model preprocessing stage to ensure balanced model training.

### 4.3 Data preprocessing

In this section, data are prepared for a subsequent phase of model training and building. To ensure that the dataset meets the model requirements and achieves accurate fitting and prediction results for both fuel consumption and carbon emissions, preprocessing for the dataset is performed, establishing the following objectives:

i *Data cleaning:* Missing, null and duplicate values in the dataset leading to bias, skewness or inaccuracies in model training and outcomes must be removed. After determining whether any entries in the data were duplicates, null or missing, they were either eliminated, in the case of duplicates, or replaced with nonnull entries, in the case of missing entries. For categorical data, missing/null entries are filled with dataset mode, whereas mean values are used for numerical data.
ii *Feature Handling, Data Transformation & Standardization:* To preserve information related to predicting consumption and carbon emissions during data preprocessing, the considered models must incorporate both numerical features and categorical features handling which can be carried out as follows:
  - Categorical feature handling: To transform categorical variables into numerical representations, one-hot encoding has been utilized for the categorical variables ‘Make’, ‘vehicle_class’, ‘Transmission’, and ‘Fuel Type’. Combined with numerical features, encoded categorical features are subsequently used by models for analysis. The resulting encoded variables are then combined with the numerical features in the dataset to form the final model input feature space. In addition, the first category of each feature is dropped to prevent multicollinearity between variables and bias.
  - Numerical feature scaling: Standardization is applied, as many regression algorithms are sensitive to scale differences between features and ensure that everyone contributes equally to the model’s performance. The numerical features were normalized via a z- score standardized scale, thereby reducing the impact of varying units and magnitudes by maintaining the data distribution shape through centering the data with a mean value of zero and standard deviation of 1. This was particularly crucial for features that have different scales, such as fuel consumption and engine size. The equation for z- scaling is as follows:

$$X_{scaled}=\frac{X-\mu }{\sigma }$$ (1)

where $X_{scaled}$ is the transformed data variable, μ is the mean and σ is the standard deviation. In this way, the normalized value of a sample X  is obtained by the difference between the original value and the average of the considered records of the attribute by the standard deviation of all samples.

iii *Data splitting:* After all the previous preprocessing stages, the original data were randomly divided into subsets for training and testing at a ratio of 80:20. This ensures reproducibility, such that consistent splitting is maintained across different program runs and prevents data leakage by ensuring that the model’s performance is assessed through unseen data.

### 4.4 Model building

Predicting fuel consumption and carbon emissions via data-driven approaches is regarded as a multivariate problem that requires regression techniques, as these numerical variables are influenced by multiple input features. These techniques, whether linear regression-based or nonlinear regression-based, use labelled data to create a model that captures the underlying relationships between features such as engine size, number of cylinders, transmission type, and fuel type and the numerical values of specific target variables—fuel consumption and CO₂ emissions. While linear regression models are known for their simplicity and low demand for computational resources, nonlinear models are typically more powerful, accurate and flexible, making them ideal for higher predictive accuracy, especially when dealing with complex, large and nonlinear relationships in the data.

To support data-driven decision-making in the context of sustainable transportation and emission reduction, this work conducts a comparative analysis of several regression techniques that are conducted for ICEV and PHEV data from Canada. The comparison includes linear, nonlinear, and ensembled ML-based models where the main objective is to identify the most effective models that are capable of capturing relationships between vehicle characteristics and their consecutive consumption and emissions. In total, thirteen different predictive algorithms (4 linear, 3 traditional nonlinear, and 6 ensemble ML) are examined in this work. Notably, the ensemble category includes three state-of-the-art techniques: XGBoost, CatBoost, and LightGBM. To increase the accuracy of the predictive models, the model hyperparameters are tuned after training via Grid SearchCV with 5-fold cross-validation. This approach allows the optimal model configuration for each algorithm to be identified through initial exploration of combinations of predefined parameters.

*Linear-Based Regression Models:*

One of the most recognized and well-known models in statistics and ML is linear regression-based models, of which four different models are investigated, including linear regression, Ridge, Lasso, and Elastic Net, which can be briefly described as follows:

a) **Linear regression (LR):** This is a baseline model indicating associations among variables by best fitting a linear equation to data such that the error difference between the actual and predicted values is minimized [62]. LR is fast and simple yet may exhibit poor generalization performance in the case of large datasets and features [63].
b) **Penalty-based** models include Ridge, Lasso and Elastic net regression models, which are often referred to as regularized versions of LR. Through the introduction of a penalty term, these types of models work better with high-dimensional data than do LR models by imposing constraints on the size of the model coefficients, referred to as shrinkage and regularization. In Lasso regression, the least absolute shrinkage and selection operator is applied, thus controlling model complexity by eliminating unnecessary features [54]. Ridge regression, on the other hand, adds a penalty to the loss function that is equivalent to the square of the magnitude of the coefficients, making it useful when there is multicollinearity between variables [64]. In contrast, Elastic Net aims to capture the strengths of both Ridge and Lasso by combining both penalties [65].

Linear regression techniques minimize the ordinary least squares loss, as specified in (2), where *n* is the total number of samples, $y_{i}$ is the observed actual value for the i-th sample, β  is the vector of model parameters, and $\left(y_{i}-{\text{x}}_{i}^{⊤}\beta \right)$ is the residual prediction error. For penalty-based models, however, this equation is extended by introducing a regularization term, as presented in (3), to control model complexity, such that λ controls the strength of regularization and Ω(β) represents the penalty function to balance model bias and variance. Ridge, Lasso, and elastic net regression apply L2 according to (4), L1 as in (5), and combined penalties in (6), such that α∈[0,1] determines the relative weighting between sparsity L1 and coefficient shrinkage L2 in the elastic net formulation.


$${min}_{\beta }\frac{\text{1}}{n}\sum_{i=\text{1}}^{n}{\left(y_{i}-x_{i}\beta \right)}^{\text{2}}$$ (2)

$${min}_{\beta }\frac{\text{1}}{n}\sum_{i=\text{1}}^{n}{\left(y_{i}-x_{i}\beta \right)}^{\text{2}}+\lambda \Omega (\beta )$$ (3)

$$\Omega (\beta )={\left\|\beta \right\|}_{\text{2}}^{\text{2}}=\sum_{j=\text{1}}^{p}\beta_{j}^{\text{2}}$$ (4)

$$\Omega (\beta )={\left\|\beta \right\|}_{\text{1}}=\sum_{j=\text{1}}^{p}\left|\beta_{j}\right|$$ (5)

$$\Omega (\beta )={\alpha \left\|\beta \right\|}_{\text{1}}+(\text{1}-\alpha ){\left\|\beta \right\|}_{\text{2}}^{\text{2}},\;\;\;\;\;\;\alpha \;\in [\text{0},\text{1}]$$ (6)

i *Nonlinear-Based Regression Models:*

In this work, 9 nonlinear ML models are investigated, including 3 traditional nonlinear ML and 6 ensemble ML models, which can be briefly described as follows:

a) **Conventional nonlinear:** This study covers 3 nonlinear ML models, including SVR, GP and DTs. SVR, which is a kernel regression technique, is recognized as one of the most robust and accurate methods among the data mining algorithms [66]. Predictions of continuous variables can be achieved by identifying the hyperplane that optimally maximizes the margin between the data points and hyperplane, producing robust predictions. Similarly, GP models are also a kernel-probabilistic strategy for learning correlations of numerous variables in the data learning set, making them particularly ideal for nonlinear forecasting problems [67]. They make predictions via kernels and offer uncertainty metrics for those predictions via the Bayesian framework [68], making them good candidates for small datasets. DT regression, on the other hand, predicts the target value of a variable by learning decision rules inferred from the input features. Using a group of logical decision rules, data are split into smaller subsets on the basis of feature values, creating a tree-like structure. Splits are chosen to minimize the variance of the target variable; thus, they are suitable for complex and nonlinear data representations [69].
b) **Ensemble ML models:** For ensemble models, both bagging and boosting algorithms are compared in this work, using GB, HGB, RF, with particular emphasis on state-of-the-art boosting architectures such as XGBoost, CatBoost, and LightGBM. Boosting strategies, such as GB, XGBoost, CatBoost and HGB, employ the core principle of sequential learning, where each new model corrects the errors of previous models [51]. GB combines weak learners of DTs to generate a strong learner that can make accurate predictions. To minimize the overall prediction error, the gradient descent algorithm is fitted on the residual errors of the previous learners in a sequential manner, thus achieving high accuracy with a customizable loss function [21]. XGBoost is a tree-based GB model that fits a decision tree to the negative gradient of the objective function in each iteration. Through the addition of a regularization penalty term, the model’s coefficients are altered, which influences the prediction outcome, making them ideal not only for handling missing data but also for faster training owing to their parallel computation capability. On the other hand, the GB approach is applied similarly to HGB but uses a histogram-based approach for binning features, where the dataset’s feature histograms are used to construct decision trees [70], thereby enhancing both computational efficiency and model performance. To better handle categorical variables, CatBoost is another ensembled algorithm specially designed for categorical boosting that improves model accuracy and robustness by mitigating overfitting through ordered boosting while avoiding target leakage during training. Unlike traditional one-hot encoding, which relies on transforming categorical variables into binary variables, CatBoost replaces categorical values with statistically derived values through target-based encoding [71], thus allowing features to be handled without the need for an extensive preprocessing phase, making it more suitable for large datasets with categorical features. Similarly, LightGBM, another gradient-boosting technique, is based on gradient-based one-sided sampling (GOSS), exclusive feature bundling (EFB), and histogram and leafwise growth. These design optimizations increase their training efficiency, enable them to consume less memory and provide predictions with high accuracy, particularly with highly dimensional datasets [72].

In contrast, bagging algorithms such as RF employ parallel learning during data training, build multiple decision trees on different subsets of the data and features, and then aggregate their predictions. This reduces both variance and bias in the model and improves the ability of the model to generalize[73]. Compared with single decision trees, predictions of the bootstrapped subsets of data are combined and averaged, making them more immune to overfitting and more robust to noise and outliers, resulting in higher predictive accuracy. Thus, these methods are well suited for datasets with large feature counts (high dimensionality) and can handle missing values in the data without requiring explicit imputation.

### 4.5 Hyperparameter tuning

To test all the algorithms effectively, all the ML models were trained via the same unified pipeline incorporating preprocessing, model definition, and hyperparameter search, following the flowchart presented in Fig 1(B). For the models where hyperparameter tuning is possible, cross-validated GridSearchCV with 5-fold validation has been implemented. Conversely, standard configurations, such as linear regression and GP, have been adopted for models without parameter grids. After the hyperparameters were determined, each algorithm was retrained using the corresponding set of best hyperparameters obtained from GridSearchCV, thus minimizing the cross-validation error [74].

### 4.6 Model evaluation

The choice of the most appropriate model with the best performance is usually driven by a trade-off between flexibility and accuracy. While linear models are inherently simple and highly interpretable, their simplicity can be a limitation when dealing with complex, nonlinear datasets, eventually affecting their predictive performance. In contrast, more complex data correlations can be captured through nonlinear ML models but at the expense of greater chances of overfitting. Combining multiple models through ensemble ML, on the other hand, offers a compromise of enhanced predictive accuracy while mitigating the shortcomings of individual linear or nonlinear models, thus offering a balanced, middle ground solution. For these reasons, this section employs a study of the previously mentioned models in an attempt to find the most suitable modelling strategy that will present the best trade-off for predicting energy consumption and carbon dioxide emissions for EVs.

To assess the effectiveness of each model along with its accuracy, consistency, and ability to predict actual values, various performance metrics are analysed. In this energy prediction problem, the performance of each ML algorithm was evaluated via three regression evaluation metrics, namely, the root mean squared error (RMSE), the mean absolute percentage error (MAPE), and the R-square ($R^{\text{2}})$, as expressed in equations (7–9).

To determine the RMSE, the total number of data points is divided by the square root of the sum of the squared deviations between the predicted and actual values. Thus, the RMSE penalizes larger errors and has the same unit as the target variable, which makes it more interpretable; however, it is sensitive to outliers [75]. Given that $\overset{―}{y}$ is the mean of the actual target values and that ${\hat{y}}_{i}$ is the model’s predicted value, the RMSE can be presented as in (7) by:


$$RMSE=\sqrt{\frac{\text{1}}{n}\sum_{i=\text{1}}^{n}{\left(y_{i}-{\hat{y}}_{i}\right)}^{\text{2}}}$$ (7)

The MAPE aims to quantify the prediction error as a percentage of the actual value of the target variable. Consequently, the error is standardized and presented in percentage values, making it comparable and interpretable across many data points, as depicted by equation (8). This is beneficial in scenarios where the target variables exhibit considerable variability and where prediction mistakes might substantially impact the overall performance of the model [76].


$$MAPE=\frac{\text{1}}{n}\sum_{i=\text{1}}^{n}\left|\frac{y_{i}-{\hat{y}}_{i}}{y_{i}}\right|\times \text{1}\text{0}\text{0}$$ (8)

R-squared, also known as the coefficient of determination, is a statistical metric for evaluating how well a model fits the actual dataset. A value between 0 and 1 is determined by dividing the model’s explained variance by the overall data variance, where a greater number denotes a better fit [77]. Given that R2 reflects how well independent variables explain the dependent variable, R^2^ is also often referred to as accuracy and can be presented as in (9).


$$R^{\text{2}}=\text{1}-\frac{\sum_{i=\text{1}}^{n}{\left(y_{i}-{\hat{y}}_{i}\right)}^{\text{2}}}{\sum_{i=\text{1}}^{n}{\left(y_{i}-\overset{―}{y}\right)}^{\text{2}}}$$ (9)

## 5. Results and performance evaluation

This section presents the results obtained from linear, nonlinear and ensembled predictive models for energy consumption and emission estimation. As previously mentioned, the data were split into training and testing sets at a ratio of 80:20, with a total of 13 models evaluated through three statistical metrics. All the models were trained and tested via the same unified pipeline presented in Fig 1 to ensure fair comparison and prevent data leakage across the experiments. Before the predictive results are presented, Table 3 summarizes the optimized hyperparameters for each model through GridSearchCV with 5-fold cross-validation for both fuel consumption and CO2 emission predictions.

**Table 3:** Tuned hyperparameters for consumption and emission models.

Model	Parameters and Values	
	Fuel Consumption	CO_2_ Emission
**Ridge Regression**	α = 0.02	α = 1.0
**Lasso Regression**	α = 0.01	α = 0.001
**ElasticNet**	α = 0.1, $L{\text{1}}_{ratio}$= 0.5	α = 0.01, $L{\text{1}}_{ratio}$= 0.8
**SVR**	C = 100, ε = 0.1, γ = 0.1	C = 10, ε = 0.1, γ = ‘scale’
**Decision Tree**	max_depth = 10, min_samples_split = 2	max_depth = 10, min_samples_split = 5
**Random Forest**	n_estimators = 300, max_depth = None	n_estimators = 100, max_depth = None
**GB**	n_estimators = 100, learning_rate = 0.2, max_depth = 3	n_estimators = 200, learning_rate = 0.1, max_depth = 5
**HistGradientBoosting**	max_iter = 200, learning_rate = 0.1, max_depth = 6	max_iter = 200, learning_rate = 0.1
**XGBoost**	n_estimators = 200, learning_rate = 0.1, max_depth = 3	n_estimators = 200, learning_rate = 0.1, max_depth = 5
**CatBoost**	iterations = 300, learning_rate = 0.2, depth = 4	iterations = 300, learning_rate = 0.1, depth = 6
**LightGBM**	n_estimators = 500, learning_rate = 0.05, num_leaves = 31, max_depth = –1	n_estimators = 300, learning_rate = 0.1, num_leaves = 31, max_depth = –1

The regularization strength in the linear and regularized regression models is controlled by the parameter λ, which is represented as α in the Python programming libraries; ε and C represent the tolerance margin and penalty parameters in SVR, respectively; γ controls the influence of individual training samples in the kernel-based methods; and the l1_ratio determines the balance between the L1 and L2 penalties in the elastic net formulation.

### 5.1 Energy consumption prediction

Table 4 compares the performance of the 13 tested models; accordingly, their predictive accuracy and robustness are evaluated by collectively assessing each model’s error magnitude, relative prediction deviation, and overall goodness-of-fit. For the linear model category, **Ridge** regression achieved the best performance compared with the other techniques, with a recorded RMSE of 0.8766, a MAPE of 6.62 and an R² value of 0.9282, followed by linear regression, with similar prediction accuracies. With respect to the nonlinear model category, the DT model performed better than the SVR and GP models did, with the lowest RMSE of 1.32923, MAPE of 3.210663, and the highest R² of 0.966042; moreover, the DT model outperformed all the linear models. For the ensemble ML category, the **RF** attained the best R² score of 0.9772 and the lowest RMSE and MAPE of 0.5455 and 3.71, respectively, indicating greater generalizability and reliability than the other ensemble ML models and superior performance among all the considered predictive models.

**Table 4:** Energy consumption prediction results.

	RMSE	MAPE	R^2^
**Linear Models**			
**Linear**	0.877684	6.631284	0.928049
**Ridge**	0.876568	6.620299	0.928232
**Lasso**	1.056877	8.565347	0.895670
**Elastic Net**	1.779575	16.649308	0.704204
**Non-Linear ML Models**			
**SVR**	1.33233	3.733448	0.868071
**GP**	3.37966	3.799606	0.966042
**DT**	1.32923	3.210663	0.966042
**Ensemble ML Models**			
**GB**	0.807223	6.367255	0.9309138
**HGB**	0.706759	5.569951	0.953345
**LightGBM**	0.648465	4.869337	0.960723
**XGBoost**	0.817420	6.488343	0.937591
**Catboost**	0.647300	5.079650	0.960865
**RF**	0.545536	3.710399	0.972202

Conclusively, **RF** stands out as the top performer among all tested algorithms, demonstrating its excellent ability to predict vehicle energy consumption with minimum prediction errors and strong prediction accuracy while capturing the variance in the data as well as verifying the supremacy of ensemble ML models over their linear and traditional nonlinear counterparts.

**Fig 5:**
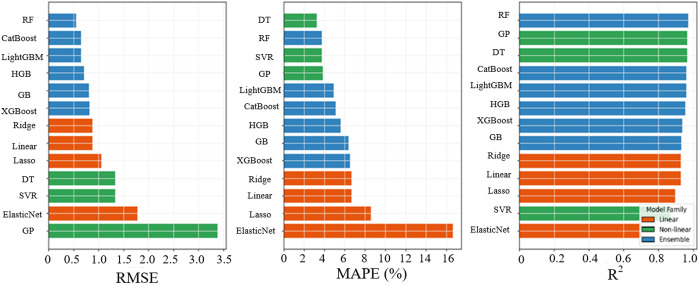
Fuel consumption performance metrics of all ML models.

For a clearer interpretation of the comparative results, Fig 5 illustrates the performance of all the evaluated models across the RMSE, MAPE, and R² metrics. The figure demonstrates the relative positioning of different model families, illustrating how ensemble-based approaches consistently cluster in regions of lower prediction error and higher fitting performance compared with linear and traditional nonlinear models. This visualization complements the quantitative results presented in Table 4 by further showing the overall performance trends rather than individual metric values, thus offering a better intuitive comparison of model robustness and predictive behaviour across all studied model categories.

**Fig 6:**
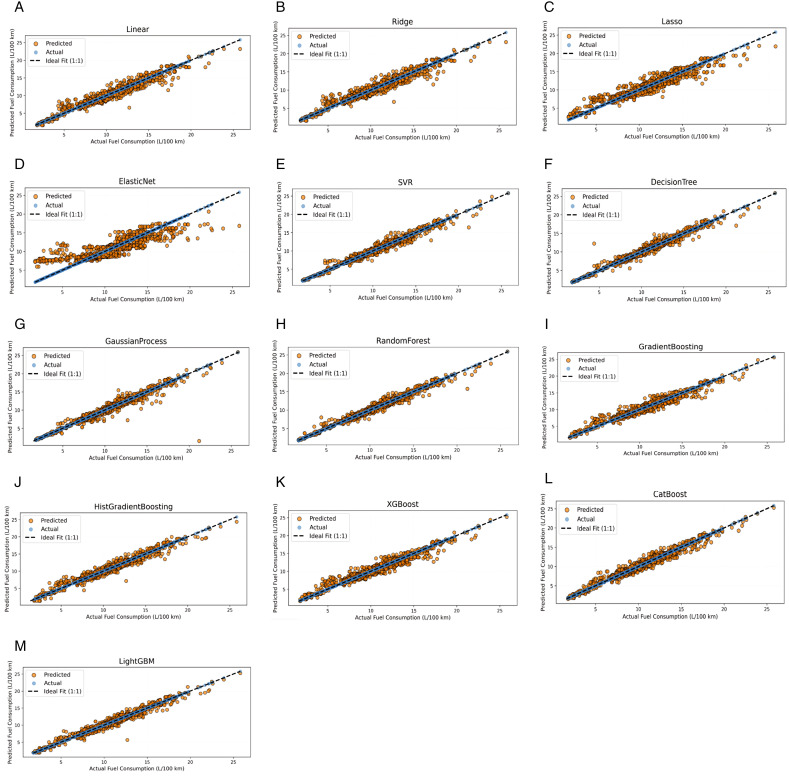
Actual versus predicted consumption plots: (A) Linear regression, (B) Ridge, (C) Lasso, (D) Elastic Net, (E) SVM, (F) DT, (G) GP, (H) RF, (I) GB, (J) HGB, (K) XGBoost, (L) CatBoost, (M) LightGBM.

In order to assess model fitting performance, a visual representation of actual versus predicted parity plots are illustrated in Fig 6, where an ideal performance when actual and predicted values align is represented by the 1:1 dashed line. For models with a high degree of accuracy, more clustering should be observed around the 1:1 line, indicating high generalizability. For linear models, performance is moderately aligned with the ideal lines but with noted widening scatter with highly consuming vehicles. Elastic net exhibits the weakest fit among all linear architectures, with increased dispersion confirming the model’s limitations in capturing nonlinear interactions in the dataset and the presence of systematic bias. For nonlinear models, including SVR, GP, and DT, tighter clustering is observed in the parity plots, especially for low- and medium-fuel-consuming vehicles employing the SVR and GP models, thereby indicating better performance capabilities than those of linear approaches. Conversely, DTs show high signs of possible overfitting, which can be observed by the very high dispersion around the ideal parity line, thus highlighting the limited model’s generalization abilities. Ensemble-based approaches clearly exhibit the most consistent parity behaviour, where GB, HSG, XGBoost, CatBoost, and LightGBM show strong predictive alignment with minor deviations in some models. Notably, CatBoost, HGB, and RF exhibit the closest adherence to the ideal 1:1 line, indicating high predictive accuracy and low systematic error.

### 5.2 CO2 emissions prediction

Table 5 shows the results of the prediction of CO_2_ emissions for all vehicle types. Again, the three previously considered statistical metrics are utilized to evaluate the performance of the tested three models’ categories, representing 13 predictive algorithms. Among all linear models, linear regression and Ridge regression are more accurate than Lasso is, with a higher R² value of approximately 0.988 and lower RMSE and MAPE values of approximately 3.6 and 1.7, respectively. Elastic net underperformed, with the highest RMSE of 14.64, MAPE of 5.384, and lowest R² value of 0.954. In contrast, the nonlinear models show better overall performance, particularly DT (RMSE of 2.205, MAPE of 1.125) and SVR (RMSE of 2.1997, MAPE of 1.342555), with R² values higher than 99% for both, indicating strong performance and better reliability than those of the GP model and verifying the superiority of the nonlinear models over the linear models. The ensemble ML models, on the other hand, stand out as having the best performance, with the RF model attaining the lowest RMSE and MAPE values of 1.6296 and 0.42937, respectively, and the highest R² value of 0.998771 among all 13 considered models.

**Table 5:** CO_2_ emission prediction results.

CO_2_ Emission Prediction Results			
	RMSE	MAPE	R^2^
**Linear Models**			
**Linear**	3.564828	1.718457	0.988646
**Ridge**	3.595379	1.682191	0.988755
**Lasso**	3.683162	1.868422	0.987142
**Elastic Net**	14.643579	5.384784	0.954692
**Non-Linear ML Models**			
**SVR**	2.199771	1.342555	0.994146
**GP**	3.754867	0.585581	0.977526
**DT**	2.205799	1.125568	0.991596
**Ensemble ML Models**			
**GB**	3.708595	1.498824	0.996596
**HGB**	1.878899	0.589221	0.998198
**LightGbM**	5.699438	1.470452	0.992824
**XGBoost**	2.635232	1.462177	0.998072
**Catboost**	2.620228	0.941574	0.998488
**RF**	**1.629604**	**0.429377**	**0.998771**

**Fig 7:**
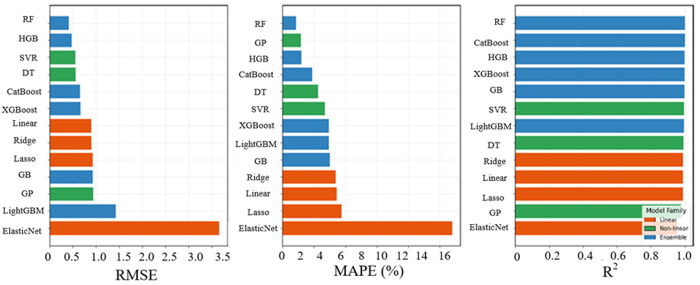
CO_2_ Emission performance metrics of all ML models.

For a better visual comparison, Fig 7 illustrates the distribution of prediction errors and goodness-of-fit metrics for the CO₂ emission estimation task across all evaluated ML models, such that the relative performance trends among different model families are clear. Similar to fuel consumption estimation, ensemble-based approaches generally achieve lower prediction errors and higher accuracy compared with linear and traditional nonlinear techniques. These findings further confirm the superiority of ensemble-based ML algorithms in terms of robustness and predictive consistency, particularly RF, which shows a consistent, top performing behaviour across both fuel consumption and CO₂ emission prediction tasks, through maintaining the lowest prediction errors and highest accuracy score.

**Fig 8:**
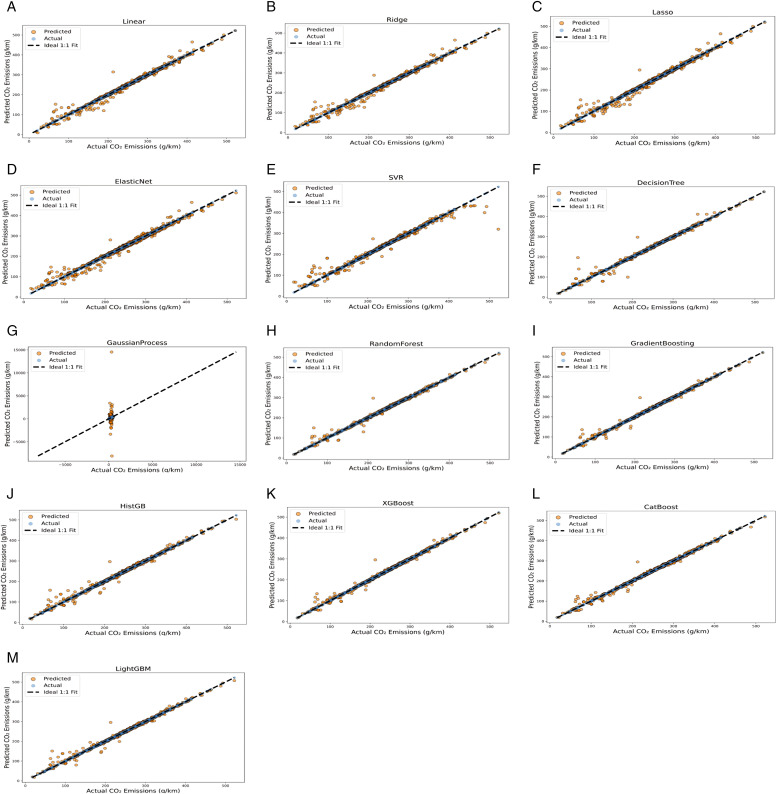
Actual versus predicted CO_2_ emissions plots: (A) Linear regression, (B) Ridge, (C) Lasso, (D) Elastic Net, (E) SVM, (F) DT, (G) GP, (H) RF, (I) GB, (J) HGB, (K) XGBoost, (L) CatBoost, (M) LightGBM.

Finally, Fig 8 shows the actual versus predicted plots for all 13 tested modelling approaches. Notably, these plots reveal solid distinctions between all model categories being studied in this analysis. While some linear models, such as Linear, Ridge and Lasso, show acceptable data alignments between the predictions and true emission values, Elastic Net clearly deviates, especially with mid- and high-range CO_2_-emitting vehicles, thus reflecting weak model generalizability. For nonlinear models, SVR achieves acceptable performance by maintaining clear clustered data scatter. In contrast, GP and DT show high dispersion and scattering, especially with high-CO_2_-emitting vehicles. In general, all the ensemble ML models outperform the linear and nonlinear models, where GB, XGBoost and CatBoost perform strongly but with slight variance, whereas HGB and RF stand out for their tight clustering, with RF showing excellent and consistent accuracy. These visual diagnostics reinforce the quantitative findings reported earlier, confirming that ensemble learning—especially RF—offers a reliable and robust framework not only for fuel consumption prediction, but also for CO₂ emission estimation by effectively maintaining stable predictive behaviour across diverse vehicle technologies.

### 5.3 Statistical significance testing

Besides visual parity plots and pointwise error calculations, it is important to assess whether the evaluated performance differences are statistically meaningful rather than data-split dependent. For this reason, statistical significance analysis was performed in this subsection, where the dataset was partitioned using an 80:20 split with proportional representation of vehicle categories, while model tuning and evaluation were further reinforced through 5-fold cross-validation to ensure robustness and eliminate any likelihood of overfitting to a particular data split. Accordingly, the mean value of each evaluation metric across the k folds was computed according to (9).


$$\overset{―}{M}=\frac{I}{K}\sum_{k=\text{1}}^{K}M_{k}$$ (9)

where $M_{k}$ represents the performance metrics obtained from k folds, where K=5 for 5-fold validation.

To verify that the best-performing model offers a statistically significant improvement over a baseline approach, formal statistical significance testing was conducted. Since the RF model managed to outperform all the other tested models for consumption and emission estimation, its performance was benchmarked against the baseline Linear regression model.

**Fig 9:**
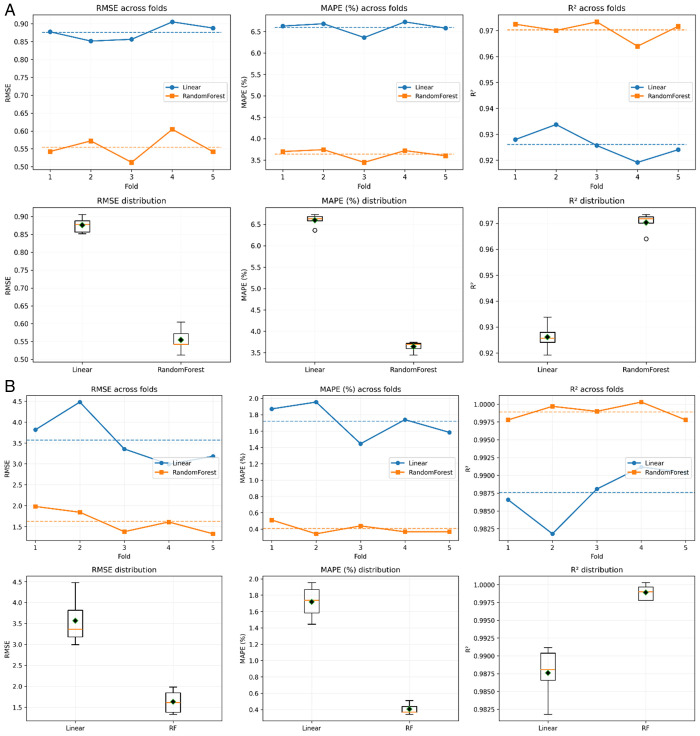
Statistical significance testing (A) Fuel consumption, (B) CO_2_ prediction.


As depicted in Fig 9(A), for consumption prediction from the 5-fold cross-validation results, the RF consistently outperforms the linear regression baseline across all evaluation metrics, scoring a lower average RMSE of 0.5548 compared with 0.8760 for the baseline linear metric, and subsequently, it is statistically significant, with a *p* value of 1.72 × 10 ⁻ ⁵. Similarly, a considerable decrease in the MAPE is exhibited in the RF model of 3.642%, whereas it is 6.597% for the linear model, corresponding to *p* = 6.17 × 10 ⁻ ⁹. With respect to the coefficient of determination, RF achieved a higher mean R² value of 0.9704 than 0.9262 for linear regression, achieving a statistically significant score of *p* = 2.93 × 10 ⁻ ⁵. Additionally, the standard deviation (STD) of each metric was computed to assess the stability of model performance across folds as (10):

$$STD=\sqrt{\frac{\text{1}}{K-\text{1}}\sum_{k=\text{1}}^{K}{\left(M_{k}-\overset{`}{M}\right)}^{\text{2}}}.$$ (10)

In addition, narrow confidence intervals were depicted across all five folds for consumption, with a 95% CI of 0.5112–0.5983 for RMSE and a 95% CI of 3.489%–3.794% for MAPE for RF, thus further confirming the stability and robustness of the RF model’s performance across all folds. These intervals correspond to low standard deviations of 0.035 for the RMSE and 0.123% for the MAPE, further confirming the stability and robustness of the RF model across all folds.

Similarly, Fig 9(B) for CO_2_ prediction shows the results of the 5-fold cross-validation process, again, with the RF outperforming the baseline Linear regressor. Statistical significance testing revealed that the mean RMSE values decreased from 3.5648 to 5.1767 for the RF model, with *p* = 2.00 × 10 ⁻ ³. In addition, similar improvements are observed in the MAPE, which decreases from 1.718% for Linear regression to 0.429% for RF, with *p* = 4.39 × 10 ⁻ ⁶). Moreover, RF achieved a higher mean coefficient of determination, with an R2 value of 0.99877, compared with 0.98760 for Linear regression and *p* = 6.32 × 10 ⁻ ⁶. Similar to the consumption estimation, the emission estimation RF model’s 95% confidence intervals further validate its robustness across the 5 folds, with a mean RMSE CI of 3.3328--5.1767 and a MAPE CI of 0.848%−1.304%, corresponding to standard deviations of 0.742% and 0.184%, respectively, thereby validating robustness across all cross-validation folds.

## 6. Discussion

Data-driven analyses contribute to national sustainability targets, especially when the data analysed are at the national scale. Accurately predicting CO₂ emissions and energy consumption from comprehensive vehicle datasets offers significant benefits for multiple stakeholders, who, on a broader scale, can gradually enhance the global shift toward low-emission mobility. Such predictive tools can provide valuable indicators for policymakers and regulatory bodies concerning the environmental impact of different vehicle technologies, enabling the formulation of more defined policies, carbon reduction incentives, and emission standards that are aligned with national climate goals. In addition, these insights are also valued by automotive manufacturers in benchmarking their vehicles against competitors, identifying key areas for design improvements, and most importantly, helping assess the real-world benefits of EV adoption.

### 6.1 Quantitative analysis between the proposed models and related work

As shown in Table 1, which is presented in Section 3, several studies have been conducted using the same data source, although they vary in terms of temporal coverage and modelling objectives. Nevertheless, the proposed approach excels in predicting both emissions and consumption rather than estimating one of them as well as depending on versatile data for both vehicle types rather than only one, which in turn increases its adaptability, scalability and generalizability. For further validation of the proposed models, a quantitative analysis is carried out, comparing the proposed results with those of their counterparts, which use the same dataset. Tables 6 and 7 provide this comparative analysis in terms of evaluation metrics (RMSE, MAPE and $R^{\text{2}}$) for forecasting CO2 emissions and energy consumption, respectively.

**Table 6:** CO2 emission prediction comparison with previous studies.

CO_2_ Emissions Prediction				
Ref	Technique	Results		
		RMSE	MAPE	%R^2^
[49]	Linear: Lasso	5.1729	1.0850	99.34
	Nonlinear ML: **SVR,** DT, KNN	3.5001	1.0315	99.71
	Ensemble ML models: RF, **GB**	3.3633	0.8854	99.70
				
[50]	Linear: Lasso, **Ridge,** Multiple linear Regression	--	--	97.96
	Nonlinear ML: SVR	--	--	97.62
	Ensemble ML models: **XGBoost,** RF	--	--	99.84
				
[51]	Regression: **Ridge**	2.3700	--	99.10
	Nonlinear ML: **SVR**	2.0300	--	99.40
	Ensemble ML models: Histogram, **Catboost**	1.9000	--	99.60
				
[52]	Linear: **Linear**, Lasso	31.0000	--	74.00
	Nonlinear: **GAMs**	28.0000	--	76.00
	Nonlinear ML: **SVR,** DT, KNN	3.5001	1.0315	99.71
	Ensemble ML models: RF, **GB**	3.3633	0.8854	99.70
				
[53]	Nonlinear ML: **SVR**, KNN	3.8600	--	99.50
	Ensemble ML model: **RF**	3.1900	--	99.70
				
[54]	Linear: Lasso**, Ridge**	4.9262	--	99.27
	Nonlinear ML: SVR, **DT,** KNN	4.4465	--	99.40
	Ensemble ML models: **XGBoost,** RF, LGBM, Hist, Gradient boost, Ada Boost	2.6554	--	99.79
	DL: CNN, DNN, GRU, RNN, LSTM, and MLP	3.8789	--	99.56
				
[55]	ANN	--	0.1935	--
				
[35]	DL: CNN	--	--	71.74
				
[56]	DL: LSTM, **Bi-LSTM**	--	0.03560	93.55
				
[57]	Nonlinear ML: **DT,** KNN, SVR,	0.0344	--	96.40
	Ensemble ML models: **RF,** Boosting, Gradient Boost, LightGBM, XGBoost	0.0308	--	97.11
	DL: GRU, LSTM, BiLSTM, CNN, MLP, BiGRU, **XMARL**	0.0118	--	99.56
				
[58]	Nonlinear ML: DT, **KNN,** SVR	0.0256	5.1800	97.94
	Ensemble ML: XGBoost, ADA boost, Catboost, **Three Combined ensemble ML models (DT, KNN,XGB)**	0.0187	3.1900	98.89
	Deep learing: BiLSTM, LSTM, **Carbon MLP**	0.0142	2.5900	99.78
				
**Proposed**	Linear: Linear, **Ridge,** Lasso, Elastic (train)	3.5954	1.6822	98.88
	Nonlinear ML: SVR, GP, **DT**	2.2058	1.1256	99.16
	Ensemble ML models: XGBoost, Hist, Catboost, gradient boost, **RF**	**1.6296**	**0.4294**	**99.88**

**Table 7:** Fuel consumption prediction comparison with previous studies.

Fuel Consumption Prediction				
Ref	Technique	Results		
		RMSE	MAPE	%R^2^
[49]	x	x	x	x
[50]	x	x	x	x
[56]	x	x	x	x
[51]	x	x	x	x
[53]	x	x	x	x
[56]	x	x	x	x
[55]	x	x	x	x
[35]	DL: CNN	x	x	70.06
[54]	x	x	x	x
[57]	x	x	x	x
[58]	x	x	x	x
**Proposed**	Linear: Linear, **Ridge,** Lasso, Elastic (train)	0.8766	6.6203	92.82
	Nonlinear ML: SVR, GP, **DT**	1.3292	3.2107	96.60
	Ensemble ML models: XGBoost, Hist, Catboost, gradient boost, **RF**	0.5455	3.7104	97.22

Almost all related works focused exclusively on CO₂ emission prediction, neglecting fuel and energy consumption, except for the work in [35], which resembles the proposed approach as the sole study to provide a comprehensive analysis covering both fuel consumption and CO₂ emission prediction. However, the study applied the CNN-based DL approach with the smallest dataset among all the methods, featuring lower accuracies, i.e., R^2^ values of 71.74% and 70%, than the proposed RF-based ensemble ML approach, with R^2^ values of 71.74% and 70% for emissions and consumption, respectively, verifying the superiority of the proposed prediction models.

Other related works that considered only carbon emissions can be categorized as follows. GHG emission prediction in [49–51,53] was achieved through the adoption of several data-driven modelling strategies via Linear regression, nonlinear regression, and ensemble ML models. Compared with other regression approaches, the latter achieves the highest accuracies; GB, XGBoost, and CatBoost, and GB, RF and XGBoost are applied in [49–51,53], as demonstrated in Table 6. Comparatively, the RF-based ensemble ML applied in the proposed approach resulted in lower RMSE and MAPE values, verifying its excellent prediction performance. On the other hand, the research conducted in [55] targeted PHEVs only for predicting CO2 emissions via artificial neural network (ANN) models, making it the only study to include PHEVs in their analysis of the dataset. Nevertheless, the study did not include ICEV vehicles, which limits the complete comparative understanding of emission performance across different vehicle technologies. On the other hand, the highest accuracies in each [54,56,58] were achieved by applying DL-based approaches, i.e., the gate recurrent unit (GRU), bidirectional long short-term memory (Bi-LSTM) and light multilayer perceptron (MLP) approaches. Despite their high accuracy values with respect to computed errors, they achieve lower R^2^ values than the proposed RF ensemble ML models do, which feature less complexity. In fact, the performance metrics for the ensemble ML model architecture were close to those of the DL models, thus reflecting that the added complexity of the DL models is not sufficiently justified by a significant improvement in prediction accuracy. Finally, both studies in [52,57] neglected categorical features, thus omitting contextual variables that could enhance the model’s predictive capabilities, resulting in the absence of class-related emission trends, potentially affecting the model’s generalizability.

Compared with related methods, the proposed predictive approach in this paper proposes generalized data-driven models for both carbon emissions and fuel consumption prediction on the basis of mixed data of both ICEVs and PHEVs. Compared with other ensemble ML models developed in the literature, the proposed RF-based approach achieves higher accuracies. Moreover, it achieves accuracies that are competitive with those of the DL models developed in related works, yet it has a less complicated realization and computational burden.

### 6.2 Proposed work contributions

A detailed comparative analysis of the model results verified the superiority of the ensemble ML-based RF models over other regression alternatives. Moreover, compared with other predictive models developed in related works that use datasets from the same source, the proposed RF-based models in this paper have the following capabilities:

- The emission and consumption predictions of the proposed models rely on two combined datasets provided by NRCan covering both ICEVs and PHEVs, unlike most related studies, which depend on their developed model for the analysis of only ICEV datasets. Hence, the proposed models are considered more versatile prediction tools for different types of light-duty vehicles.
- Rather than predicting only vehicle emissions in related works, the proposed work developed two models, one to predict emissions and the other to predict fuel consumption, which adds to its significance in vehicle design and policymaking.
- Compared with the ensemble ML models derived in related works, the approach proposed in this paper achieves higher accuracies, verifying their superiority. Moreover, compared with other alternatives that apply DL, the proposed ensemble ML models can capture both linear and nonlinear relationships with competing accuracies without relying on overly complex architectures.

Consequently, this study provides a robust foundation for developing evidence-based environmental and vehicle design policies in any region on the basis of similar relative datasets, paving the path for industries to adapt and innovate to anticipate economic and environmental impacts. This serves well the increasing commitment to the United Nations’ Sustainable Development Goals (SDGs), particularly those focused on climate action, clean affordable energy and sustainable urban development.

### 6.3 Real-time deployment and policy simulation considerations

The development of an accurate and reliable model for EV energy consumption and emissions is a fundamental requirement, not only for vehicle-level control but also for making EV policies and regulations, infrastructure planning, and policy simulation. In practical deployment scenarios, such models must meet additional challenges related to computational efficiency, hardware compatibility, and scalability, particularly when integrated into real-time vehicle systems or used for extensive fleet-level and regulatory analyses.

Despite recent advances in data-driven modelling, deploying predictive models in real-time vehicle systems and large-scale policy simulations is influenced by a wide range of interacting factors, including vehicle dynamics, road conditions, weather, auxiliary loads, traffic conditions, and driving styles [78]. In real-time applications, due to the strict delay constraint of data communication, instantaneous predictions are often required for decision-making, which results in stringent computational constraints and hardware feasibility [79]. While ensemble-based ML models offer improved accuracy and robustness, their real-time deployment in embedded vehicle systems must balance prediction fidelity against runtime efficiency.

With respect to fleet-level analyses and policy simulations, scalability and interpretability are critical considerations. Model transparency and adaptability to evolving vehicle technologies and operating conditions are essential, particularly since they rely on offline or batch processing. These considerations impose certain limitations on the current study, particularly when it transitions from offline model development to real-world operational environments and policy-driven use cases.

### 6.4 Future perspectives

With respect to future perspectives, this work could be extended to the following:

i *Fuel cell vehicles (FCVs)*

Fuel cell vehicles (FCVs) represent a vital pathway toward large-scale zero-emission mobility, particularly because of their high efficiency and clean hydrogen-based operation. However, accurate parameter estimation and robust lifespan prediction of PEM fuel cell stacks are essential for ensuring reliable performance under real-world dynamic conditions [80]. These capabilities enable improved energy management, enhanced durability assessment, and more effective control strategies.

Therefore, extending future work to include FCV systems—supported by advanced estimation and degradation-prediction methods—will significantly contribute to the development of sustainable, hydrogen-powered transportation solutions.

ii *Hybrid ML–optimization architectures*

The current machine learning–based forecasting framework can be extended by integrating advanced optimization techniques to further increase the prediction accuracy and support decision-making [81]. By accurately estimating system parameters, metaheuristic optimization approaches enable predictive ML models to generate reliable forecasts under varying driving cycles and operating conditions. Moreover, these algorithms can be integrated with HEV predictive frameworks to develop energy management strategies to minimize fuel usage, support the design of low-emission control policies, and contribute to sustainable transportation goals.

Hence, future work can explore hybrid ML–optimization architectures to automatically tune model parameters, identify optimal energy-management strategies, and generate more robust emission-minimization scenarios under varying driving conditions. Additionally, future studies may investigate the real-time implementation of these hybrid models, evaluate their performance on larger and more diverse datasets, and assess their applicability across different vehicle platforms to improve scalability and generalizability.

## 7. Conclusion

This work presents an ensemble ML-based framework for predicting fuel consumption and carbon emissions in light-duty combustion and hybrid vehicles through the fusion of two mixed datasets for ICEVs and PHEVs into a unified data-driven modelling approach. Through a comprehensive comparison against 13 ML models ranging from linear to nonlinear and state-of-the-art approaches, the RF model consistently demonstrates superior predictive accuracy and robustness by outperforming other traditional and nonlinear regressors. Additionally, when benchmarked with state-of-the-art approaches, including CatBoost, LightGBM and XGBoost, the RF still stands out as the top performer, thereby indicating the effective handling of complex and nonlinear relationships within the datasets. Compared with DL approaches, it achieves competitive performance that lies in maintaining simpler implementation and reduced computational efforts, thereby highlighting its practical applicability with tabular and moderate-sized datasets. Compared with previous methods, the proposed framework focuses on predicting two targets rather than on a single target, thus providing a more generalizable and robust predictive tool that may be useful in energy planning and sustainable vehicle design. Nevertheless, this work could be extended to explore FCVs for achieving zero emission, in addition to studying hybrid ML-optimization techniques to improve forecasting accuracy and enable effective minimization of fuel consumption and emissions in HEVs.
